# mTORC1 inhibitors rapamycin and everolimus as host-directed therapy for tuberculosis

**DOI:** 10.1128/iai.00544-25

**Published:** 2026-01-21

**Authors:** Robin H. G. A. van den Biggelaar, Tom H. M. Ottenhoff

**Affiliations:** 1 Leiden University Center for Infectious Diseases, Leiden University Medical Center 4501, Leiden, the Netherlands; University of California Merced, Merced, California, USA

**Keywords:** rapamycin, everolimus, *Mycobacterium tuberculosis*, mTOR inhibitors, host-directed therapy

## LETTER

Li et al. recently reviewed advances in host-directed therapy (HDT) for tuberculosis (1). Special attention was given to autophagy-targeting strategies including inhibitors of the mammalian target of rapamycin (mTOR). Autophagy allows macrophages to recapture mycobacteria that have escaped from phagosomes and redirect them to lysosomes for degradation. Induction of autophagy by starvation or high concentrations of mTOR inhibitor rapamycin (i.e., sirolimus) suppressed the intracellular mycobacterial survival in infected macrophages (2). In addition, rapamycin reduced lung inflammation in a mouse model of tuberculosis during the chronic phase of infection (3). These observations highlight mTOR inhibition as an interesting strategy for HDT.

Li et al. noted that rapamycin incompletely inhibits mTOR complex (mTORC)1 and does not target mTORC2, potentially triggering feedback activation (1). They further highlight a study by Andersson et al., in which sole inhibition of mTORC1 by rapamycin enhanced *Mycobacterium* (*M*.) *tuberculosis* replication in human immunodeficiency virus (HIV) co-infected macrophages (4). Consequently, the authors proposed that future studies should focus on dual mTORC1/2 inhibitors, of which everolimus was incorrectly given as an example. Indeed, rapamycin is widely regarded as a potent and effective inhibitor that inhibits many, but not all functions of mTORC1 at nanomolar concentrations (5). However, the same is true for everolimus, a rapamycin analog (6). Both drugs selectively target mTORC1 through the formation of an inhibitory complex with FK506 (tacrolimus)-binding protein 12 (FKBP12), which does not interact with mTORC2, while they differ in their pharmacokinetic properties.

The risk of increased mycobacterial survival following mTORC1 inhibition by rapamycin treatment in HIV co-infected macrophages is indeed concerning (4). However, Andersson et al. also demonstrated that Torin-1, an ATP-competitive mTOR kinase inhibitor, similarly increased *M. tuberculosis* replication, showing that dual mTORC1/2 inhibition does not necessarily prevent this risk.

Finally, Li et al. mention that everolimus has been reported to show direct antimicrobial activity against *M. tuberculosis*, based on a study by Ashley et al., in which near-therapeutic nanomolar concentrations were used (7). Most studies, however, have reported direct antimicrobial activities only at supraphysiological micromolar concentrations (8–11). Clinically relevant nanomolar concentrations of everolimus have reduced *M. tuberculosis* survival in infected macrophages (12), supporting host-directed mechanisms, but not in peripheral blood mononuclear cells (10). Whether rapamycin and everolimus act against *M. tuberculosis* at therapeutic concentrations remains to be validated (10).

Everolimus has been evaluated in a phase II clinical trial for pulmonary tuberculosis, where it improved lung function (13), while dual mTORC1/2 inhibition reduced relapse in mice (9). Thus, mTOR inhibitors are promising HDT candidates, perhaps more for immunomodulation than direct or host-directed antimicrobial effects. At the same time, caution is warranted for potentially worse outcomes during HIV co-infection or other comorbidities (4, 14).

## EDITOR'S NOTE

**Ed. Note**: The authors of the original article did not respond at the time of publication.
